# Component‐resolved diagnosis using guinea‐pig allergens elucidates allergen sensitization profiles in allergy to furry animals

**DOI:** 10.1111/cea.13873

**Published:** 2021-04-09

**Authors:** Kyra Swiontek, Stéphanie Kler, Christiane Lehners, Markus Ollert, François Hentges, Christiane Hilger

**Affiliations:** ^1^ Department of Infection and Immunity Luxembourg Institute of Health Esch‐sur‐Alzette Luxembourg; ^2^ National Unit of Immunology‐Allergology, Centre Hospitalier Luxembourg Luxembourg; ^3^ Department of Dermatology and Allergy Center Odense Research Center for Anaphylaxis University of Southern Denmark Odense Denmark

**Keywords:** allergen, Guinea‐pig, IgE‐diagnosis, lipocalin

## Abstract

**Background:**

Furry animals are an important source of indoor allergens. Diagnosis of allergy to small pets such as guinea‐pigs still relies on animal dander extracts which do not allow to define the primary sensitization source.

**Objective:**

To identify major guinea‐pig allergens and to evaluate their potential as marker allergens for in vitro IgE‐diagnosis in comparison with dander extracts.

**Methods:**

A group of patients allergic to guinea‐pig (*n* = 29) and a group of patients allergic to cat and dog (*n* = 30) were recruited for the study. A panel of four guinea‐pig lipocalin allergens was expressed as recombinant proteins in *E*. *coli*. Specific IgE were quantified by ImmunoCAP and ELISA.

**Results:**

The combination of 4 guinea‐pig lipocalin allergens, including 2 new lipocalins, Cav p 1.0201 and Cav p 6.0101, and the previously characterized lipocalins Cav p 2 and Cav p 3, enabled the identification of 90% of all patients allergic to guinea‐pig. The vast majority had specific IgE to Cav p 1 (83%). Cav p 6 shares 54% sequence identity with Fel d 4 and Can f 6 and was found to be IgE‐cross‐reactive with these allergens. In the group of cat‐ and dog‐allergic patients, 73% had also specific IgE to guinea‐pig dander. However, only 27% of the cat /dog‐allergic patients had specific IgE to any of the non‐cross‐reactive guinea‐pig allergens Cav p 1, Cav p 2 or Cav p 3. The high prevalence of IgE to guinea‐pig dander could be explained by IgE‐cross‐reactivity among serum albumins and certain lipocalins.

**Conclusions and clinical relevance:**

The availability of specific allergen markers is essential for the assessment of primary sensitization, especially in polysensitized patients. The proposed panel of guinea‐pig allergens Cav p 1, Cav p 2 and Cav p 3 is a first step to component‐resolved IgE‐diagnosis of allergy to small furry pets.

## Graphical abstract

Component resolved diagnosis and the availability of specific marker allergens (right) allows the determination of primary sensitization in patients which show multiple sensitizations to animal dander extracts (left).

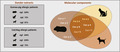

## 1 INTRODUCTION

In western life‐style countries, pet dander is a major cause of inhalant allergy and sensitization to furry animals is a major risk factor for developing allergic asthma and rhinitis. ^1^, ^2^, ^3^ Cats and dogs are the main allergen sources, but numerous small furry animals, kept nowadays as pets, gain more and more importance.^4^, ^5^ Among laboratory animal workers, occupational allergy to rabbits and rodents is highly prevalent.^6^ The popularity of small furry pets including rodents such as guinea‐pigs will also increase allergen exposure in the domestic environment.^7^ As pet allergens are ubiquitous and found in public places, sensitization can also occur without direct animal contact.^8^

Major and minor cat and dog allergens have been extensively characterized allowing to distinguish genuine IgE‐sensitization from mere IgE‐cross‐reactivity between certain cat and dog allergens.^7^ A reliable distinctive element is sensitization to a specific marker allergen such as the major cat allergen Fel d 1.^9^ On the contrary, sensitization to a member of the cross‐reacting family of serum albumins, or to the Fel d 4/Can f 6/Equ c 1 lipocalin subgroup will often give positive results with several animal dander extracts and may be wrongly interpreted as true primary sensitization.^10^, ^11^, ^12^ For small furry pets, many allergens remain undefined and in vivo and in vitro diagnosis commonly relies on allergen extracts.^13^ This may lead to inaccurate clinical diagnosis due to IgE‐cross‐reactivity between certain cat, dog and small pet allergens and vice‐versa, especially in polysensitized individuals.

The objective of the present study was to provide specific marker allergens for the diagnosis of allergic sensitization to guinea‐pig, a pet with a high sensitizing power.^6^ Four guinea‐pig lipocalin allergens including 2 new recombinant allergens were assessed for their diagnostic value in a cohort of guinea‐pig‐allergic patients and then used to analyse potential cross‐reactivities in a cohort of allergic patients sensitized to both cat and dog.

## 2 MATERIALS AND METHODS

### 2.1 Patient recruitment

The first patient cohort consisted of 29 patients (mean age = 38.5 years, 79% female) exposed to guinea‐pig dander with a history of inhalant allergy. Of these, 26 were doubtlessly only allergic to guinea‐pigs while 3 had possibly also symptoms to other pets. All were sensitized to guinea‐pig as shown by positive skin prick tests and the presence of specific IgE (sIgE) to guinea‐pig extract. The second patient cohort consisted of 30 patients (mean age = 35.0 years, 37% female) with inhalant allergy in presence of cat or dog or cat and dog and having positive skin tests and specific IgE to both cat and dog. These patients had no exposure to guinea pigs. Both cohorts were recruited at the National Unit of Immunology‐Allergology at the Centre Hospitalier de Luxembourg. Skin prick tests solutions were from Stallergenes (Anthony Cedex, France), and in vitro IgE measurements to guinea‐pig, cat and dog epithelium were performed with ImmunoCAP (ThermoFisher Scientific). Ethical approval was obtained from the National Committee for Medical Research Ethics (No°201001/06 and No 201307/04), and informed consent was obtained for all subjects.

### 2.2 Establishment of a panel of guinea‐pig allergens.

Two guinea‐pig lipocalin allergens, Cav p 2 and Cav p 3, have been previously characterized in detail.^14^ Additional allergens were identified by IgE‐immunoblot of guinea‐pig hair extracts using guinea‐pig‐allergic patient sera and N‐terminal sequencing of IgE‐reactive bands as described in Supplementary Methods. Two new lipocalins, named Cav p 1.0201 and Cav p 6.0101 by the WHO/IUIS allergen nomenclature subcommittee, were purified from guinea‐pig hair by ion exchange chromatography. Corresponding cDNAs were cloned from guinea‐pig harderian gland and expressed in *E*. *coli*. Cav p 4, guinea‐pig serum albumin, was purchased from Sigma‐Aldrich. Polyclonal antibodies were raised against recombinant Cav p 1 and Cav p 6. Mice were immunized with 50 μg of allergen suspended in Al(OH)_3_ (Sigma‐Aldrich), and 3 boosts were administered subsequently at 3‐week intervals.^14^ Animal handling met the European guidelines for experimental animals and the internal regulations of the institute.

### 2.3 Quantification of specific IgE to recombinant and native allergen components

Specific IgE (sIgE) to recombinant Cav p 1, Cav p 2, Cav p 3, Cav p 6 and to native Cav p 4 (serum albumin), as well as recombinant Fel d 4 and Can f 6, were measured by ELISA as described.^15^

### 2.4 Statistical analyses

sIgE frequencies to individual components in the two cohorts were compared using 2x2 contigency tables and Fishers's exact test using GraphPad Prism 8. The Venn diagram summarizing the sensitization profiles of guinea‐pig‐allergic patients was constructed by using an online tool.^16^

## 3 RESULTS

### 3.1 Isolation and characterization of Cav p 1 and Cav p 6

**FIGURE 1:**
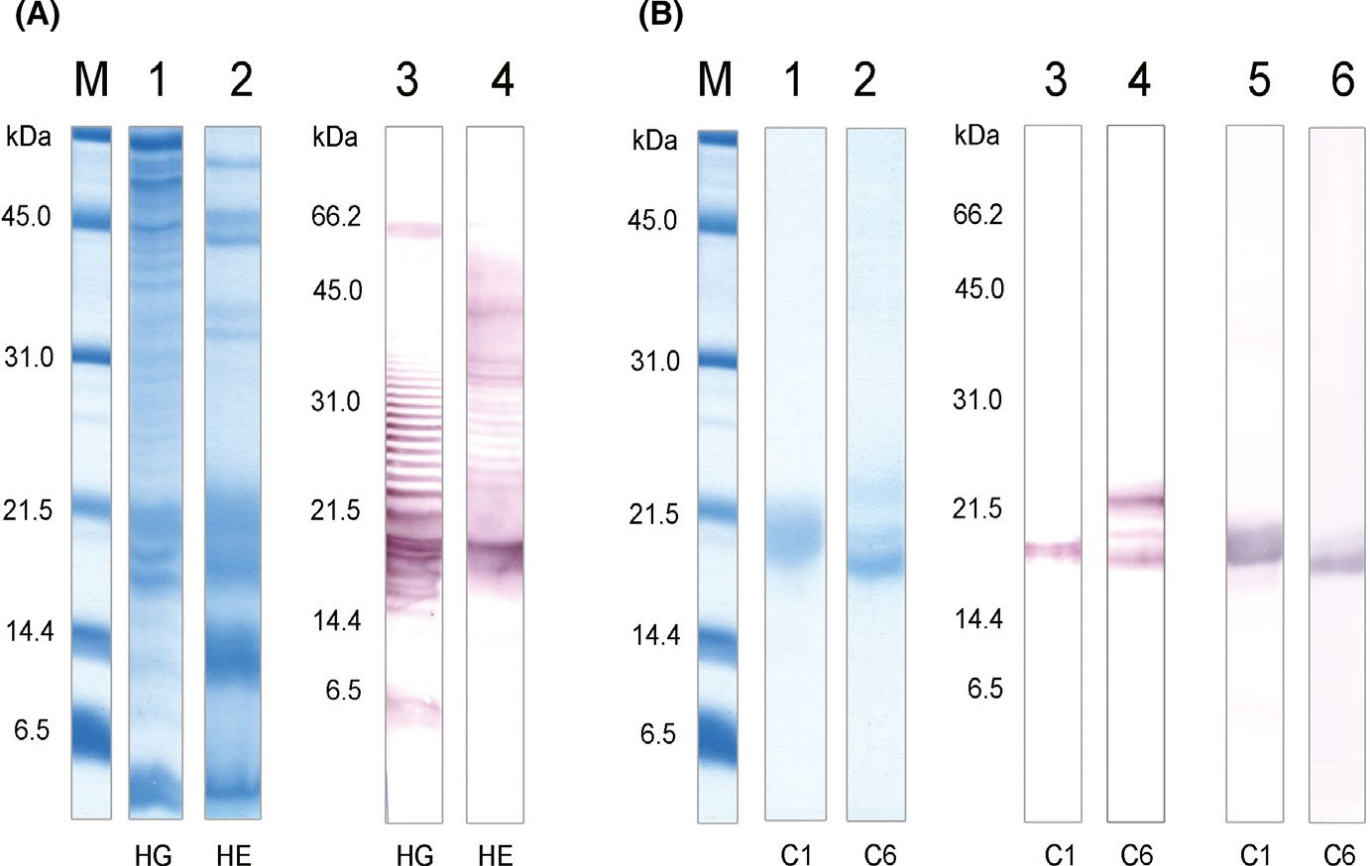
Detection and purification of IgE‐reactive proteins from guinea‐pig. (A) Proteins were extracted from guinea‐pig harderian gland (HG) and hair (HE), separated by SDS‐PAGE and stained with Coomassie (lanes 1 and 2) and immunoblotted using a representative patient serum (no GP‐15, lanes 3 and 4). (B) Cav p 1 (C1) and Cav p 6 (C6) were isolated from guinea‐pig hair extract, separated by SDS‐PAGE, stained with Coomassie (lanes 1 and 2) and immunoblotted with patient serum (no GP‐15, lanes 3 and 4) or with polyclonal mouse serum raised against rCav p 1 (lane 5) or rCav p 6 (lane 6). M, molecular weight marker

IgE‐reactive bands were detected in hair and harderian gland extract from guinea‐pig using sera of guinea‐pig‐allergic patients and the corresponding proteins were purified from hair by ion exchange chromatography (Figure 1). As lipocalins tend to have highly similar molecular weights and isoelectric points, it is not possible to purify them to homogeneity under native conditions. Additional bands observed in Figure 1B, (lanes 2 and 4) are due to other IgE‐reactive proteins. Polyclonal mouse serum directed against recombinant Cav p 6 recognizes only the lower and major of the 3 bands (Figure 1B, lane 6). The N‐terminal sequences of the 2 isolated IgE‐binding proteins were determined by Edman degradation. The sequence of the first protein, S(E/Q)I(N/S)GDWNTIALSADNKEKIEEG, is identical to an allergen named Cav p 1, previously described as major allergen in guinea‐pig hair and urine by Fahlbusch et al.^17^ Only 15 N‐terminal residues had been determined at that time and the allergen had not been fully characterized. The sequence identified in the present study has two ambiguous aa positions, pointing to the existence of putative isoforms. A third ambiguous position was found at position 8 (N/D) on Cav p 1 purified from the harderian gland (data not shown).

**FIGURE 2:**
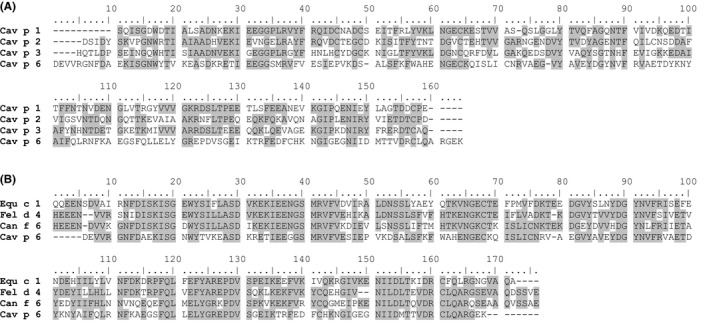
Sequence alignment of guinea‐pig lipocalins and of mammalian lipocalins with high sequence similarity to Cav p 6. (A) Sequence alignments of guinea‐pig lipocalins Cav p 1 (A0A48HRI4), Cav p 2 (P83508), Cav p 3 (F0UZ12) and Cav p 6 (S0BDX9). (B) Guinea‐pig Cav p 6 was aligned with horse Equ c 1 (Q95182), cat Fel d 4 (Q5VFH6) and dog Can f 6 (H2B3G5) using Clustal W. Residues identical between at least 2 lipocalins are shaded in grey

The cDNA corresponding to allergen Cav p 1, named Cav p 1.0201, has now been cloned and the sequence clearly classifies it as lipocalin. Sequence identity is 44% to Cav p 2, 46% to Cav p 3 and 29% to Cav p 6 (Figure 2A). Other mammalian allergens with highest identity are the Siberian and golden hamster lipocalin allergens Phod s 1 and Mes a 1 (43%).

The N‐terminal amino acid sequence of the second protein, DEVVRGNFDAEKISG, relates to a newly identified allergen named Cav p 6 by the WHO/IUIS allergen nomenclature subcommittee. It also belongs to the lipocalin family, and it shows a relatively high amino acid sequence identity to a group of lipocalins that have been shown to be cross‐reactive: Fel d 4 (54%), Can f 6 (54%) and Equ c 1 (49%) (Figure 2B).^10^, ^11^

All 4 lipocalin allergens, Cav p 1, Cav p 2, Cav p 3 and Cav p 6, were detected in guinea‐pig hair protein extracts (Figure S1A). The anti‐Cav p 3 mouse serum gave a strong signal in saliva, whereas Cav p 1 and Cav p 6 were absent, and Cav p 2 was detectable as faint band (Figure S1B). Cav p 1, Cav p 2 and Cav p 6 were also found in the harderian gland (Figure S1C).

### 3.2 Allergen‐component‐resolved IgE‐profile of guinea‐pig‐allergic patients

**FIGURE 3:**
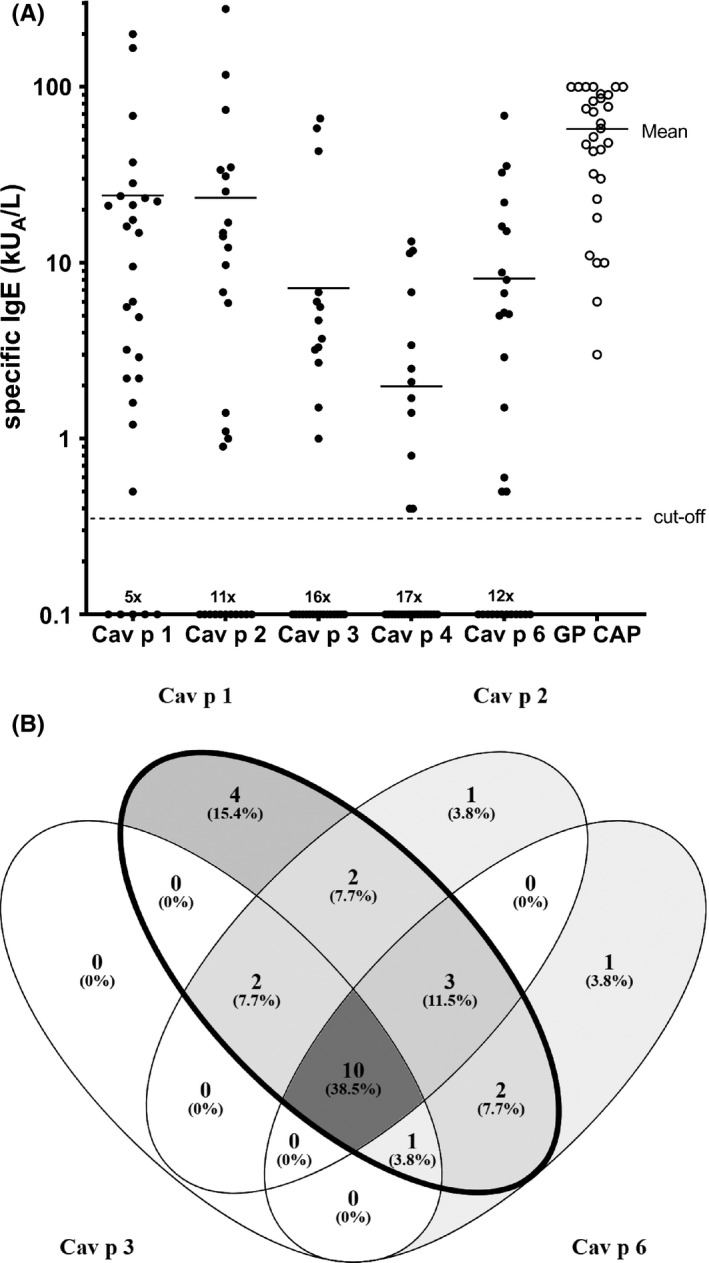
Detection of specific IgE to guinea‐pig allergens in a cohort of guinea‐pig‐allergic patients. (A) IgE‐binding to recombinant Cav p 1, Cav p 2, Cav p 3, Cav p 4 and Cav p 6 was quantified by ELISA, binding to guinea‐pig dander was determined by ImmunoCAP. (B) Venn diagram summarizing the sensitization profiles of guinea‐pig‐allergic patients

To assess the diagnostic value of the 4 guinea‐pig allergens, a group of patients allergic to guinea‐pig was screened for the presence of sIgE to the 4 lipocalins. Twenty‐four of 29 (83%) guinea‐pig‐allergic patients had sIgE to rCav p 1 (Figure 3A). rCav p 2 was recognized by 62% (18/29), rCav p 3 by 45% (13/29) and rCav p 6 by 59% (17/29) of the patient sera. Forty‐one percent of the sera presented sIgE binding to guinea‐pig serum albumin, Cav p 4. In total, we were able to detect 26 out of 29 (90%) guinea‐pig‐allergic patients using the 4 recombinant guinea‐pig allergens. Most patients were sensitized to several guinea‐pig lipocalin allergens and only 2 of these patients did not have sIgE to Cav p 1 (Figure 3B). Three other patients did not show IgE‐reactivity to any of the 4 lipocalin components tested. One of these 3 patients had sIgE to Cav p 4. The 2 other patient sera were clearly positive with guinea‐pig extract in ImmunoCAP, pointing to the existence of one or several other unknown guinea‐pig allergens.

**FIGURE 4:**
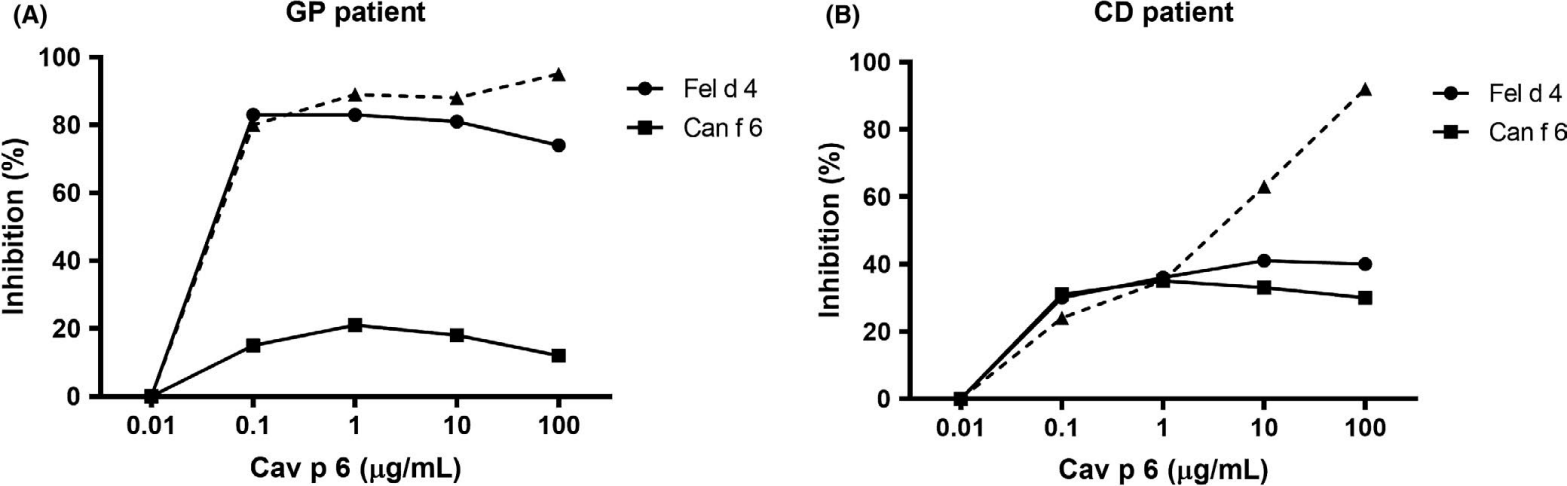
ELISA IgE‐inhibition. IgE‐reactivity to Cav p 6, Fel d 4 and Can f 6 was inhibited with increasing amounts of Cav p 6 in a guinea‐pig‐allergic patient (GP‐04) (A), and a cat‐ and dog‐allergic patient (CD‐31) (B). Dotted line: self‐inhibition by Cav p 6

As Cav p 6 shares relevant sequence identity with Fel d 4 and Can f 6, we assessed a potential IgE‐cross‐reactivity to these lipocalins. Sera with sIgE to Cav p 6 were tested for the presence of IgE reacting to Can f 6 and Fel d 4. Thirteen out of 17 (76%) Cav p 6 positive patients were also positive for Fel d 4, and 5 (29%) with Can f 6 (Table S1). All except one of these patients had higher IgE titers to Cav p 6 than to the homologous Fel d 4 or Can f 6, suggesting that the major cross‐reactivity was from guinea‐pig to cat and/or dog. A representative example of cross‐reactivity is shown for patient GP‐04 (Figure 4A). This patient is allergic to guinea‐pig (sIgE >100 kU_A_/L), but she is also sensitized to cat (sIgE 4 kU_A_/L) and dog (sIgE 28 kU_A_/L). Inhibition with increasing doses of Cav p 6 inhibited IgE‐binding to Fel d 4 up to 83%, binding to Can f 6 up to 21%. The results point to a cross‐sensitization from guinea‐pig to cat and probably to a co‐sensitization to dog, although she does not have a dog.

### 3.3 IgE‐reactivity of cat/dog‐allergic patients to guinea‐pig components

**FIGURE 5:**
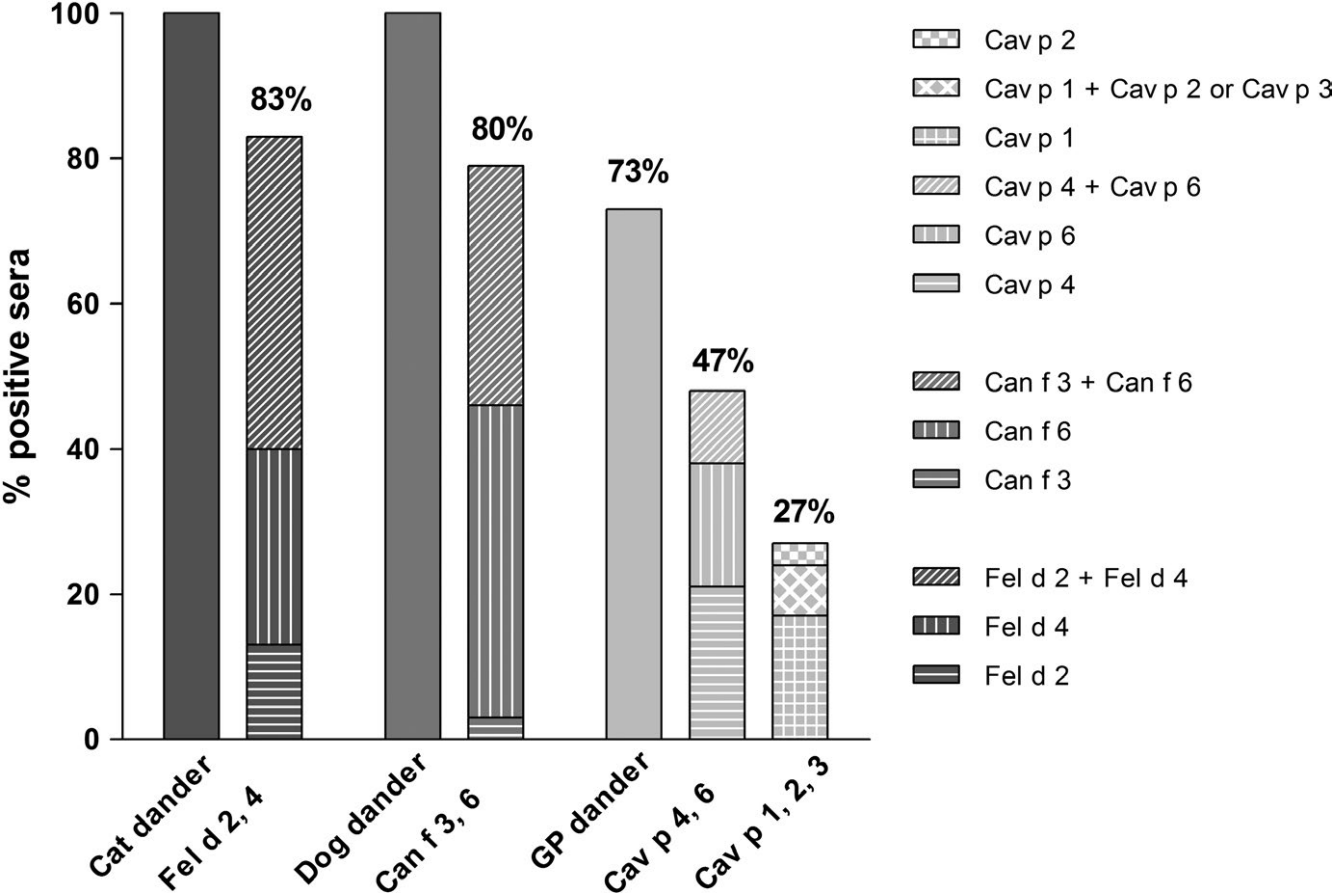
Detection of specific IgE to allergens of cat, dog and guinea‐pig in a cohort of patients allergic to cats and dogs. IgE‐reactivity was measured by ImmunoCAP (cat, dog and guinea‐pig (GP) dander, Fel d 2 and Can f 3) and by ELISA (recombinant Fel d 4, Can f 6, Cav p 1, Cav p 2, Cav p 3, Cav p 6, native Cav p 4). Percent values are based on the entire patient cohort

In order to assess the specificity, respectively, the cross‐reactivity of guinea‐pig components, a cohort of 30 patients allergic to cat/dog (Table S2) and with no exposure to guinea pigs were tested for the presence of sIgE to guinea‐pig dander and allergen components (Figure 5, Figure S2). In this cohort, a high percentage had sIgE to cross‐reactive cat allergens Fel d 2/Fel d 4 (83%) or to the corresponding cross‐reactive dog allergens Can f 3/Can f 6 (80%).

Twenty‐two of the 30 cat and dog sensitized patients (73%) had a positive IgE test to guinea‐pig epithelium, titers between 0.42 and 51.6 kU_A_/L. A majority of these sera reacted to the cross‐reactive molecules Cav p 4 and/or Cav p 6: fourteen out of 22 sera were positive for Cav p 4 and/or Cav p 6. Only 7 sera reacted to Cav p 1 (titers 0.6–3.4 kU_A_/L), and 2 of these were also positive for Cav p 2, resp. Cav p 3. One other patient had specific IgE to Cav p 2, but not Cav p 1. The frequencies of IgE positive sera to Cav p 1, Cav p 2 and Cav p 3 in both patient cohorts are significantly different (Fisher's exact test, *p* < 0.0001, <0.0001 and <0.0002, respectively), indicating that all 3 lipocalins can be considered as markers for primary sensitization to guinea‐pig. The presence of sIgE to Cav p 6 was also more frequent in the guinea‐pig‐allergic cohort, but the difference was less significant (*p *= .0182).

The remaining 8 patients were positive to guinea‐pig dander, but negative when analysed for IgE‐reactivity to Cav p 4 and the 4 guinea‐pig lipocalins, pointing possibly to the existence of another cross‐reactive allergen in guinea‐pig dander.

IgE‐cross‐reactivity among Fel d 4, Can f 6 and Cav p 6 lipocalin subfamily members was further assessed by ELISA inhibition using a cat‐ and dog‐allergic patient serum (CD‐31) (Figure 4B). IgE‐binding to Fel d 4 and Can f 6 could only partially be inhibited by Cav p 6 (41% and 35%, respectively). This patient was initially diagnosed as guinea‐pig sensitized, but it turned out that he was sensitized to several furry animals (sIgE cat and dog >100 kU_A_/L, sIgE guinea‐pig 23 kU_A_/L) and that, according to the inhibition results with Cav p 6, his sensitization to guinea‐pig is a cross‐sensitization from cat and dog.

## 4 DISCUSSION

Animal dander extracts are still widely used for in vivo and in vitro allergy diagnosis. Although they have several drawbacks, such as variable allergen content and the presence of cross‐reactive molecules, they constitute a sensitive screening tool for the detection of sensitization to animals and they are often used as first step in diagnosis.^7^ In a second step, it is important to determine the primary allergen sensitization source in order to give patients optimal advice on animal avoidance and immunotherapy.

In the present study, we have used four guinea‐pig lipocalins and serum albumin to analyse the IgE‐reactivity profile of a group of guinea‐pig‐allergic patients.

A partially characterized 20 kDa protein named Cav p 1 had been purified from hair by Fahlbusch et al. and shown to be a major allergen of the guinea‐pig.^17^ In the present study, we have isolated an allergen corresponding to the N‐terminal peptide initially described. As the present sequence deviates by 3 amino acid positions from the previously published sequence, it was classified as isoallergen and named Cav p 1.0201. The cDNA coding for Cav p 1.0201 was cloned and expressed in *E*. *coli*. Another lipocalin, Cav p 6, was identified as allergen present in guinea‐pig hair, cloned and further characterized. Together with the previously identified lipocalins Cav p 2 and Cav p 3, the 2 new allergens were evaluated for their use in component‐resolved diagnosis.

Cav p 1 is the most important major guinea‐pig allergen. It is recognized by 83% of guinea‐pig‐exposed and guinea‐pig‐allergic persons. Sequence identity with other known pet lipocalins is below 47%, and Cav p 1 is thus unlikely to present significant IgE‐cross‐reactivity with other known allergens.^18^

Cav p 6 on the contrary belongs to the cross‐reactive Fel d 4, Can f 6, Equ c 1 lipocalin subfamily.^18^ Sequence identities are highest between cat Fel d 4 and dog Can f 6 (67%), identity to Cav p 6 is lower (54%). This is also reflected by the frequency of double and triple positive IgE‐tests in the group of cat/dog‐allergic patients. Whereas 70 and 80% have sIgE against Fel d 4 and Can f 6, respectively, only 27% react to Cav p 6.

In the selected cohort of cat/dog‐allergic patients, 73% were also positive with guinea‐pig dander. Eight of these 22 patients (27%) were positive, although weakly (between 0.6 and 3.4 kU_A_/L), to Cav p 1, Cav p 2 and/or Cav p 3, even though anamnesis did not report contact with guinea‐pigs. An explanation could be that these pet‐allergy‐prone individuals had contact with guinea‐pig allergens without knowing it. Other explanations could be that they did not remember previous contacts or that they omitted to report exposure to guinea‐pigs. As animal allergens are ubiquitous, this is certainly a weakness of the study. In an optimal setting, patients would need to be followed longitudinally, with detailed questionnaires and exposure monitoring, in order to assess objective exposure to animal allergens. However, at this point it can also not be excluded that there exists a weak cross‐reactivity between Cav p 1 and other pet allergens, possibly not yet defined lipocalins. Nevertheless, the presence of specific IgE to Cav p 1 is highly indicative of a primary sensitization to guinea‐pig and not to cross‐sensitization to cat and/or dog allergens (*p* < .0001).

For 14 cat‐ and dog‐allergic patients, guinea‐pig extract positivity could be explained by IgE‐cross‐reactivity either to Cav p 6 and/or Cav p 4, an assumption supported by a stronger IgE‐positivity in the cat and dog homologous molecules (Table S2). Guinea‐pig serum albumin has been shown to have a high protein sequence identity (70%–75%) with other mammalian pet serum albumins and to present a strong IgE‐cross‐reactivity within the serum albumins protein family.^12^

However, eight patients do not have specific IgE to any of the recombinant or native guinea‐pig allergens tested. Although the tested panel is quite extensive, it does probably not include all guinea‐pig allergens as suggested by the results in the primarily guinea‐pig‐allergic cohort, where 10% of the patients could not be diagnosed by using the 4 lipocalins.

In conclusion, a panel of 4 allergens was able to detect 90% of guinea‐pig‐allergic patients. Cav p 1 is the most important guinea‐pig allergen detecting by itself primary sensitization to guinea‐pig in 83% of the patients of our cohort. The prevalence of IgE directed to the panel of 3 marker allergens in a cohort of cat/dog‐allergic patients is reduced to 27% compared to 73% when using hair extracts, underlining the urgent need of marker allergens to distinguish polysensitization from cross‐reactivity.

Direct and indirect exposure to different pet animals hampers clinical diagnosis of the primary sensitizing source, but also clinical co‐sensitization is difficult to assess without the availability of marker allergens. Molecular components are essential to clarify the sensitization profile of those patients to give an accurate and in‐depth diagnosis and advice.

As shown by the analysis of the IgE‐positivity to whole guinea‐pig extract in a selected cohort of cat/dog‐allergic patients, a majority of them do not have a primary sensitization to guinea‐pig allergens. This is a strong argument for the good performance of component‐resolved diagnosis in establishing primary allergy to guinea‐pigs, especially in a context of pet polysensitization. It is also an incentive for the need of further characterisation of other small pet allergens in order to find specific marker allergens for each species and to enable a correct diagnosis in matter of allergies to furry animals.

## CONFLICT OF INTEREST

None of the authors has declared a conflict of interest related to this study.

## AUTHOR CONTRIBUTIONS

CH and KS were involved in all stages of the project, analysed the data and wrote the manuscript; KS and SK performed the experiments; FH and CL recruited patients and participated in data analysis and interpretation; MO contributed to the interpretation of data and revised the final version of the manuscript. All authors critically revised the manuscript.

## Supporting information

Supplementary Material

Fig S1

Fig S2

Table S1‐S2

## Data Availability

All data generated or analysed during this study are included in this published article and its supplementary information files.
