# Supplementary material for: Component‐resolved diagnosis using guinea‐pig allergens elucidates allergen sensitization profiles in allergy to furry animals
Source: Clin Exp Allergy. 2021 Apr 9;51(6):829–35. doi: 10.1111/cea.13873 (PMC8251889; doi:10.1111/cea.13873)
Supplement: Supplementary file 1 — Supplementary Material [file CEA-51-829-s001.docx]

**Supplemental methods**

**S1 *Source material, extract preparation and allergen purification***

Hair was cut using scissors, then extracted with phosphate-buffer-saline (PBS) containing protease inhibitors (Roche, Mannheim). Dander was obtained from Allergon (Ängelholm, Sweden).

Hair extract was dialyzed against 20mM TRIS-HCl, pH 8 and separated by anion exchange chromatography (RESOURCE Q column, GE Healthcare, Buckinghamshire, UK). Bound proteins were eluted by using a linear gradient of 0-500 mM NaCl in 20mM TRIS-HCl, pH8. Eluted fractions were analyzed by SDS-PAGE followed by silver staining, than by immunoblotting with patient serum as described.^1^ Individual fractions containing purified IgE-reactive proteins were selected for N-terminal sequencing. Fractions containing Cav p 6 were pooled and further purified by a second ion exchange chromatography (Mono Q, GE Healthcare, Buckinghamshire, UK) using a gradient of 0-500 mM NaCl in 20 mM Piperazine, pH 5.5. Individual fractions were selected for N-terminal sequencing.

Harderian gland was excised from male Dunkin Hartley guinea pigs immediately after sacrifice. For protein extraction, the tissue was frozen in liquid nitrogen, ground to powder with mortar and pestle, and suspended in Single Detergent Lysis buffer (50mM Tris-HCl pH 8, 150mM NaCl, 1% Triton X-100, protease inhibitor cocktail (Roche, Manheim, Germany). Solid material was discarded by centrifugation and the supernatant was concentrated with an Ultrafree concentrator (cut-off 5000 MW, Millipore, Bedford, Mass.). All extractions were performed on material isolated and kept at -80°C during an earlier study.^1^ Animal handling met the European guidelines for experimental animals and the internal regulations of the institute.

**S2** *Protein sequencing*

The N-terminal amino acid sequences of IgE-reactive proteins were determined by automated Edman degradation on PROCISE 49X HT Protein sequencer (Applied Biosystems, Foster City, CA) as described.^2^

**S3** *SDS-PAGE and detection of specific IgE antibodies by immunoblot*

The different extracts and purified proteins were analysed by SDS-PAGE gels under reducing conditions. Proteins were detected with Coomassie stain (GelCode Blue Stain reagent, Pierce, Rockford, IL) or silver stain (SilverSNAP stain kit II, Pierce). For immunoblotting, proteins were transferred onto a polyvinylidene difluoride (PVDF) membrane (Millipore, Bedford, Mas, USA) as described.^2^

PVDF membranes were incubated overnight with patient sera diluted 1:5 in blocking buffer or mouse anti-sera (1/5000) raised against guinea pig proteins. Bound IgE were detected with anti-human IgE antibodies labelled with alkaline phosphatase (Southern Biotech, Birmingham, Ala., USA). Bound mouse IgG were detected with anti-mouse IgG antibodies labelled with alkaline phosphatase (Sigma, Overijse, Belgium). Blots were developed by addition of nitro blue tetrazolium/5-bromo-4-chloro-3-indolyl-phosphate (NBT/BCIP) (Promega, Madison, WI).

***S4*** *RNA extraction and cloning*

Total RNA was extracted from the harderian gland using the RNeasy lipid tissue midi kit (Qiagen, Hilden, Germany) according to the manufacturer’s instructions.

cDNA was obtained according to the conditions and materials of BD SMART RACE cDNA amplification kit (Clontech-Takara, St-Germain-en-Laye, France). 3’ and 5’ RACE was performed with harderian gland total RNA. Degenerate primers were designed based on the N-terminal sequences obtained for Cav p 1 and Cav p 6. In case of Cav p 1, it was not possible to amplify cDNA by 3’RACE and a strategy amplifying a short internal fragment had to be adopted. These primers were based on a long N-terminal sequence obtained from a protein purified from the harderian gland (SQ/EIS/NGDWD/NTIALSADNIEKIEEGGPLxLYFRQIDDNADDS xITFRRYVK) Primers MQ2 GGN GAY TGG RAC ACR ATT GC and MQ7rev TT RTC RTC DAT YTG RCG GAA amplified a first 95 bp fragment. Sequences obtained from this fragment allowed to design specific forward and reverse primers for amplification of the 3’ and 5’end (Forward primer CTCTGTCTGC TGACAACAAA GAGRAGATCG AAGAGG; reverse primer CGTTTCTTCG GGTGTCAGAG AGTCTC). For Cav p 6, the primers designed on the N-terminal amino acid sequence (DEVLYGNFDAEKISG) successfully amplified Cav p 6 cDNA by 3’ and 5’RACE (Forward primer GAR AAG ATT TCR GGV AAY TGG TAT; reverse primer CTG GTT CTC GGC CAT AGA GCT C). After final sequence analysis, specific primers were used for cloning the open reading frame of both cDNAs into the expression vector pQE60 (Qiagen).

**S5** *Expression and purification of recombinant Cavia porcellus* *allergens*

The cDNA coding sequence for the mature proteins Cav p 1 and Cav p 6 were subcloned into pQE-60 (Qiagen) and expressed in *E.coli* Rosetta-gami cells (Merck, Darmstadt, Germany) as recombinant proteins with a C-terminal Hexa-Histidine tag. The recombinant proteins were purified by affinity chromatography (HisTrap^TM^ HP, GE Healthcare, Buckinghamshire, UK) under native conditions according to the instructions of the manufacturer. Recombinant proteins were further purified by ion exchange chromatography using a RESOURCE Q column (GE Healthcare) Bound proteins were eluted by a linear gradient of 0-500 mM NaCl in 20mM TRIS-HCl, pH8 and eluted fractions were dialyzed against PBS. The purity of the recombinant proteins was analysed by SDS-PAGE and silver staining. Identity was confirmed by N-terminal sequencing.

**S7** *ELISA and ELISA inhibition*

Specific IgE to recombinant allergens were quantified as described.^2,3^ A group of 22 allergic patients with specific IgE to pollen and/or mite, but not to animal dander (total IgE ranging from 16 to 370 kU/L) served as controls. Values of less than 0.35 kU_A_/L were considered negative. A standard curve was created using serial dilutions of serum from a cat-allergic patient with a known titer of sIgE against cat serum albumin Fel d 2, determined by ImmunoCAP (ThermoFisher Scientific, Uppsala, Sweden). This serum was added to wells coated with Fel d 2 and cold water fish gelatin was used as blocking agent. A standard curve was plotted for converting optical density units into specific IgE dilutions and unknown sIgE titers to animal allergens were calculated based on this standard curve.ELISA inhibitions were done by adding increasing amounts of allergen to the diluted patient serum. Sera were incubated for 2 hrs at room temperature before adding to the plate.

**References**

1. Hilger C, Swiontek K, Kler S, Diederich C, Lehners C, Vogel L, et al. Evaluation of two new recombinant guinea-pig lipocalins, Cav p 2 and Cav p 3, in the diagnosis of guinea-pig allergy. Clin Exp Allergy. 2011;41(6):899-908.
2. Hilger C, Dubey VP, Lentz D, Davril C, Revets D, Muller CP, et al. Male-specific submaxillary gland protein, a lipocalin allergen of the golden hamster, differs from the lipocalin allergens of Siberian and Roborovski dwarf hamsters. *Int Arch Allergy Immunol* 2015;**166**:30-40.
3. Kuehn A, Hilger C, Lehners-Weber C, Codreanu-Morel F, Morisset M, Metz-Favre C, et al. Identification of enolases and aldolases as important fish allergens in cod, salmon and tuna: component resolved diagnosis using parvalbumin and the new allergens. Clin Exp Allergy 2013;43:811-22.

**Supplementary figure legends**

**Figure S1:** Detection of guinea-pig allergens in guinea-pig hair extract, saliva and harderian gland. Polyclonal mouse sera raised against recombinant Cav p 1 (1), Cav p 2 (2) , Cav p 3 (3) and Cav p 6 (4) detect immunoreactive protein bands in guinea-pig hair extract (A), saliva (B) and harderian gland (C). M, molecular weight marker (kDa); lane 5, negative control.

**Figure S2**: Detection of specific IgE to guinea-pig allergens in a cohort of cat and dog allergic patients. IgE-binding to recombinant Cav p 1, Cav p 2, Cav p 3, Cav p 4 and Cav p 6 was quantified by ELISA, binding to guinea-pig dander was determined by ImmunoCAP.
