# Supplementary figures and images for: Component‐resolved diagnosis using guinea‐pig allergens elucidates allergen sensitization profiles in allergy to furry animals
Source: Clin Exp Allergy. 2021 Apr 9;51(6):829–35. doi: 10.1111/cea.13873 (PMC8251889; doi:10.1111/cea.13873)

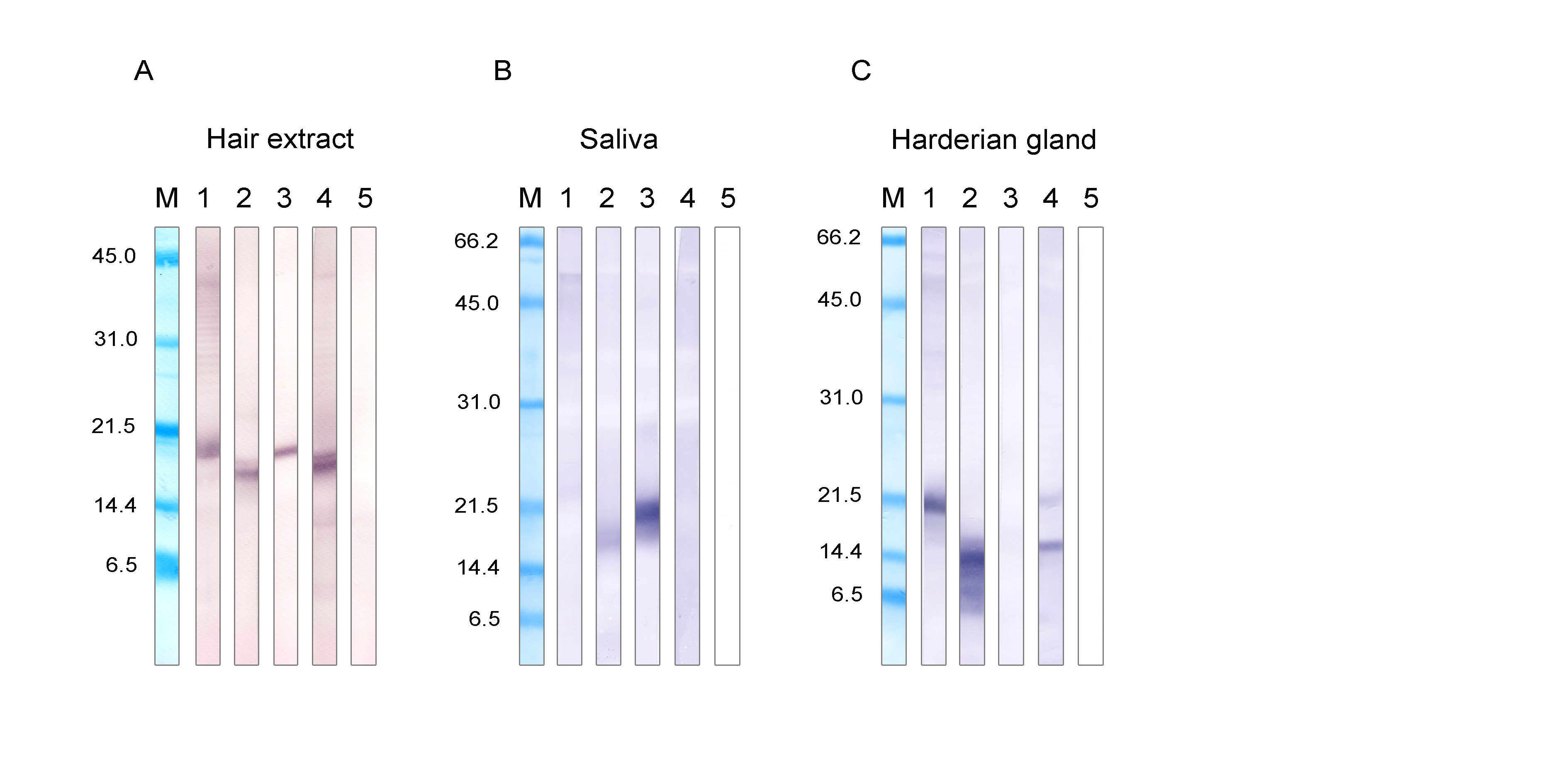

Supplement: Supplementary file 2 — Fig S1 [file CEA-51-829-s002.jpg]

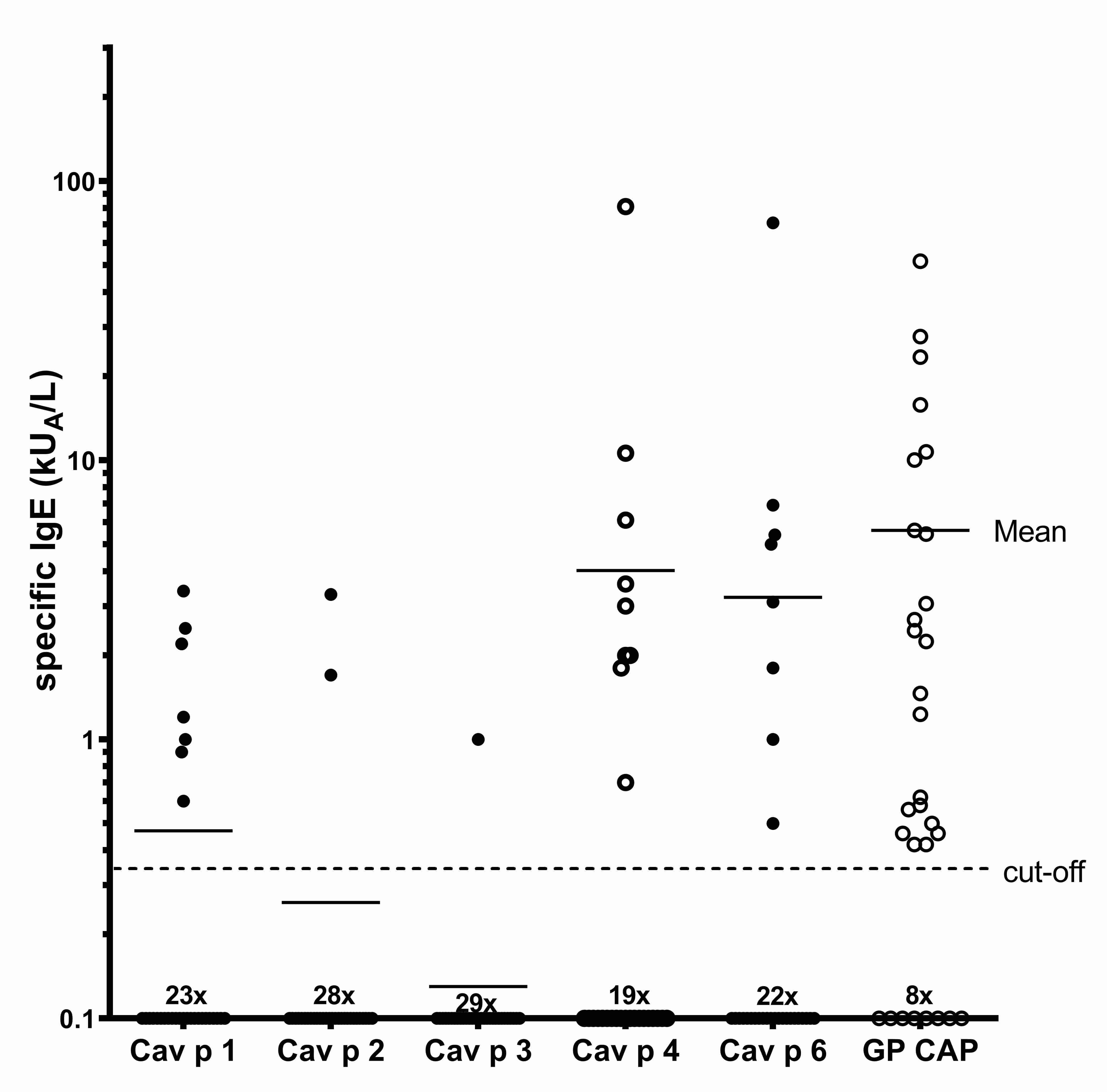

Supplement: Supplementary file 3 — Fig S2 [file CEA-51-829-s004.jpg]
