# Supplementary material for: Component‐resolved diagnosis using guinea‐pig allergens elucidates allergen sensitization profiles in allergy to furry animals
Source: Clin Exp Allergy. 2021 Apr 9;51(6):829–35. doi: 10.1111/cea.13873 (PMC8251889; doi:10.1111/cea.13873)
Supplement: Supplementary file 4 — Table S1‐S2 [file CEA-51-829-s003.pdf]

Online Table 1 : Guinea-pig allergic patient cohort

Patient code	Age (years)	Gender	Symptoms upon exposure	Exposure	GP dander e6 (kU$_A$/L)	nCav p 4 (kU$_A$/L)	rCav p 1 (kU$_A$/L)	rCav p 2 (kU$_A$/L)	rCav p 3 (kU$_A$/L)	rCav p 6 (kU$_A$/L)	Cat dander e1 (kU$_A$/L)	rFel d 4 (kU$_A$/L)	Dog dander e5 (kU$_A$/L)	rCan f 6 (kU$_A$/L)
GP-01	51	F	A, R	gp	43	0.4	0.5	0.0	0.0	0.5	<0.35	0.0	1.54	0.0
GP-02	40	F	A, R	gp	>100	1.4	166.1	276.9	0.0	5.1	21	2.7	48	0.0
GP-03	24	F	R	gp, cat (-,+), rabbit (-,-)	58	2.1	23.3	6.8	2.7	5.0	4.02	0.5	9.14	0.0
GP-04	34	F	A	gp	>100	13.2	68.4	74.0	66.0	68.6	4.33	8.7	28	6.7
GP-05	19	F	R	gp	18	1.7	0.0	0.0	0.0	0.0	1.65	0.0	0.94	0.0
GP-06	23	F	R, C	gp	72	3.4	14.8	14.8	5.6	15.1	1.45	1.8	0.84	0.0
GP-07	41	F	A	gp	47	2.5	6.0	0.0	3.2	35.5	<0.35	2.4	<0.35	0.0
GP-08	28	F	A, R	gp	86	0.4	21.3	31.0	0.0	0.0	<0.35	0.0	1.88	0.6
GP-09	46	F	A, R	gp	>100	0.0	199.5	117.0	58.2	0.6	nd	nd	0.39	nd
GP-10	28	F	A	gp, cat (-,+)	52	11.3	2.2	0.0	0.0	0.5	<0.35	1.1	<0.35	0.0
GP-11	32	F	A	gp	48	0.0	37.2	12.2	6.8	0.0	<0.35	0.5	<0.35	0.4
GP-12	47	F	A, R	gp,cat (-,+)	77	0.8	1.2	0.0	0.0	0.0	7.38	0.0	0.788	0.0
GP-13	40	M	R, C	gp	75	0.0	17.5	33.7	1.0	32.5	<0.35	nd	21.1	nd
GP-14	50	M	A, R, C	gp	90	0.0	16.1	16.9	0.0	8.0	0.55	3.6	3.99	2.4
GP-15	45	F	A, R, C	gp, cat , dog	>100	6.8	23.9	9.7	4.7	5.2	24	5.5	8.2	1.0
GP-16	15	M	A, R, U	gp, cat (-,+), dog (-,+)	44	0.0	0.0	0.0	0.0	6.7	0.48	1.8	0.67	0.0
GP-17	47	F	A	gp	91	0.0	21.1	25.4	43.1	16.1	9.27	4.7	3.27	0.0
GP-18	28	M	A	gp	23	0.0	5.6	0.0	0.0	0.0	4.93	0.0	3.59	0.0
GP-19	35	F	A	gp, cat	62	0.0	22.3	0.0	0.0	0.0	10.6	0.0	2.07	0.0
GP-20	48	M	A, R, E	gp	>100	0.0	28.3	34.9	0.0	22.0	nd	6.0	nd	0.0
GP-21	70	F	A	gp	>100	0.0	4.9	5.9	6.0	2.9	nd	0.0	nd	0.0
GP-22	38	F	A, R, C	gp, cat*	30	0.0	9.5	14.1	3.7	0.0	8	0.0	nd	0.0
GP-23	46	M	R	gp, dog	83	11.7	3.2	0.9	3.3	8.8	5.55	4.8	25	2.7
GP-24	38	F	A, R	cat, gp*, dog*	11	0.0	1.6	0.0	0.0	0.0	>100	0.0	20	1

GP-25	38	F	A, R	4 gp outside	10	0.0	0.0	0.0	0.0	0.0	2.9	0.0	0.66	0.0
GP-26	28	F	A, E, R, C	gp	10	0.0	0.0	1.1	0.0	0.0	3.99	0.0	10	1.2
GP-27	45	F	R	(gp), cat (-,+)	6	0.0	2.2	1.0	0.0	0.0	0.39	0.0	0.44	0.0
GP-28	45	F		gp, dog (-,+)	3	0.0	0.0	0.0	0.0	0.0	<0.35	0.0	0.61	0.0
GP-29	48	F	A	dog, (gp)	32	0.0	2.9	1.4	1.5	1.5	5.3	2.0	5.15	0.8
**Positive /Total**					29/29	12/29	24/29	18/29	13/29	17/29	19/26	14/27	20/23	9/27
**Percent**					100%	41%	83%	62%	45%	59%	73%	52%	87%	33%
**Mean**	38.5				58	1.9	24.1	23.4	7.1	8.1	8.3	1.7	7.5	0.6
**Median**	40.0				58	0.0	6.0	1.4	0.0	0.6	3.4	0.5	2.0	0.0

Specific IgE values are in kU_A/L and were determined by ImmunoCAP (guinea-pig (GP), cat and dog dander) and by ELISA (recombinant Cav p 1, Cav p 2, Cav p 3 and Cav p 6, native Cav p 4). CAP values ≥ 0.35 were considered positive. ELISA values less than 0.35 were considered negative and set to zero.

A, asthma; R, rhinitis; C, conjunctivitis; E, eczema; U, contact urticaria; nd, not done; gp, guinea pig.

* means intermittent ongoing exposure with symptoms and positive tests

(animal) in parentheses means previous (historic) exposure with symptoms and still positive tests

(-,-) means exposure but no symptoms and negative tests (prick and sIgE to extract)

(-,+) means exposure but no symptoms and positive tests (prick and sIgE to extract)

Online Table 2 : Cat/dog allergic patient cohort

Patient code	Age (years)	Gender	Symptoms upon exposure	Exposure	Cat dander e1 (kU_A/L)	nFel d 2 e220 (kU_A/L)	rFel d 4 (kU_A/L)	Dog dander e5 (kU_A/L)	nCan f 3 e221 (kU_A/L)	rCan f 6 (kU_A/L)	GP dander e6 (kU_A/L)	nCav p 4 (kU_A/L)	rCav p 1 (kU_A/L)	rCav p 2 (kU_A/L)	rCav p 3 (kU_A/L)	rCav p 6 (kU_A/L)
CD-01	45	F	A	dog	9.34	5.3	0.0	61	13	1.1	0.46	2.0	0.0	0.0	0.0	0.0
CD-02	22	M	E	cat, dog, rb (-,-)	>100	1.2	43	20	0.11	5.1	2.68	0.0	0.0	0.0	0.0	0.0
CD-03	32	M	A	cat, dog	85	66.4	2.2	>100	>100	51	27.7	10.6	2.2	0.0	0.0	0.0
CD-04	49	M	A	dog, (cat, fer)	76	11.6	0.7	88	19.3	5.6	10.7	1.8	3.4	0.0	0.0	0.0
CD-05	33	F	A	(dog, cat)	15	<0.1	0.0	6.23	<0.1	0.0	0.2	0.0	0.0	0.0	0.0	0.0
CD-06	56	M	R	cat, dog	73	0.6	0.0	15	<0.1	0.0	1.23	0.0	0.0	0.0	0.0	0.0
CD-07	20	F	E, A	dog	98	13	16.3	>100	17	75	51.6	3.0	0.9	1.7	0.0	5.4
CD-08	30	M	R, C	cat, dog	21	<0.1	0.0	13	<0.1	0.0	<0.1	0.0	0.0	0.0	0.0	0.0
CD-09	50	M	A	cat, dog	1.86	0.2	1.8	64	<0.1	152	0.56	0.0	1.2	0.0	0.0	0.5
CD-10	51	F	R, A	dog	2.71	0.7	0.4	18	2.53	0.5	<0.1	0.7	0.0	0.0	0.0	0.0
CD-11	19	M	R, E	cat, dog	26	<0.1	0.0	4.48		0.15	0.58	0.0	0.0	0.0	0.0	0.0
CD-12	24	M	R, C, A	cat, dog, rb (-,-), h (-,-)	78	<0.1	0.0	18	<0.1	3.3	2.45	0.0	0.0	3.3	0.0	0.0
CD-13	44	M	R	dog	70	10.2	0.0	13	6.65	0.0	1.46	3.6	0.0	0.0	0.0	0.0
CD-14	26	F	R, C, A	cat	95	<0.1	15.7	7.15	<0.1	0.8	<0.1	0.0	0.0	0.0	0.0	0.0
CD-15	17	M	R, A	cat, dog	4.86	0.6	0.0	5.47	0.32	0.0	10.02	0.0	0.0	0.0	0.0	0.0
CD-16	23	F	E	cat, dog	>100	<0.1	0.0	19	<0.1	0.5	0.46	0.0	0.0	0.0	0.0	0.0
CD-18	51	M	A	cat, dog	11	<0.1	1.4	17	<0.1	1.7	0.22	0.0	0.0	0.0	0.0	0.0
CD-19	54	F	R, A	cat, dog	>100	0.7	23	8.76	<0.1	2	0.42	0.0	0.6	0.0	1.0	1.8
CD-20	18	M	A	cat, dog	86	<0.1	48	80	<0.1	36	5.6	nd	2.5	0.0	0.0	3.1
CD-21	27	F	R, C, A	dog	1.86	<0.1	0.5	2.97	<0.1	0.9	<0.1	0.0	0.0	0.0	0.0	0.0
CD-22	30	F	A	cat, dog, cow	>100	<0.1	51	9.02	<0.1	18	2.24	0.0	0.0	0.0	0.0	5.0
CD-23	31	F	R	dog	2.54	1.3	3	24	3.85	3.6	0.24	nd	0.0	0.0	0.0	0.0
CD-24	17	M	R	cat, dog	34	18.8	4.2	5.96	11.5	0.8	3.06	6.1	0.0	0.0	0.0	0.0

CD-25	53	M	A	dog, h (-,-)	65	0.7	45	68	0.21	33	0.42	0.0	0.0	0.0	0.0	0.0
CD-26	37	F	A	cat, dog	12	0.4	0.4	1.16	<0.1	1.2	<0.1	0.0	0.0	0.0	0.0	0.0
CD-27	42	M	A	dog	1.46	<0.1	3.1	2.85	<0.1	3.7	0.5	0.0	0.0	0.0	0.0	0.0
CD-28	42	M	A	cat, dog	63	<0.1	31	17	<0.1	16.5	0.62	0.0	0.0	0.0	0.0	6.9
CD-29	21	M	A, R, E	cat, dog	>100	2.6	11.3	38	2.28	4.2	15.78	0.0	0.0	0.0	0.0	0.0
CD-30	62	M	A	cat, dog	>100	>100	24	>100	>100	12.8	5.44	81.0	0.0	0.0	0.0	1.0
CD-31	23	M	A	dog	>100	13.7	63.1	>100	14.6	55.7	23.4	2.0	1.0	0.0	0.0	70.8
**Positive/Total**					30/30	17/30	21/30	30/30	11/29	24/30	22/30	9/28	7/30	2/30	1/30	8/30
**Percent**					100%	57%	70%	100%	38%	80%	73%	32%	23%	7%	3%	27%
**Mean**	35.0				54	8.3	13.0	34.2	10.0	16.2	5.6	4.0	0.4	0.2	0	3.2
**Median**	31.0				67.5	0.6	2.0	17.5	0.0	2.65	0.6	0.0	0.0	0.0	0.0	0.0

Cat and dog allergic patient cohort. Specific IgE values are in kU_A/L and were determined by ImmunoCAP (guinea-pig (GP), cat and dog dander, Fel d 2 and Can f 3) and by ELISA (recombinant Cav p 1, Cav p 2, Cav p 3 and Cav p 6, native Cav p 4). CAP values ≥ 0.35 were considered positive. ELISA values less than 0.35 were considered negative and set to zero.

A, asthma; R, rhinitis; C, conjunctivitis; E, eczema; nd, not done; gp, guinea pig; fer, ferret; rb, rabbit; rt, rat; h, hamster.

(-,-) means exposure but no symptoms and negative tests ; (-,+) means no symptoms, but positive tests (prick and sIgE to extract)
